# Supplementary material for: Role and mechanism of NCAPD3 in promoting malignant behaviors in gastric cancer
Source: Front Pharmacol. 2024 Apr 22;15:1341039. doi: 10.3389/fphar.2024.1341039 (PMC11070777; doi:10.3389/fphar.2024.1341039)
Supplement: Supplementary file 11 [file DataSheet2.ZIP › GSEA/GO_CC/my_analysis.Gsea.1599461408869/GO_PERINUCLEAR_REGION_OF_CYTOPLASM.html]

Details for gene set GO\_PERINUCLEAR\_REGION\_OF\_CYTOPLASM[GSEA]

|  || Dataset | filtered\_dataset.sample\_info.cls#WT\_versus\_NCAPD3\_MUT |
| Phenotype | sample\_info.cls#WT\_versus\_NCAPD3\_MUT |
| Upregulated in class | NCAPD3\_MUT |
| GeneSet | GO\_PERINUCLEAR\_REGION\_OF\_CYTOPLASM |
| Enrichment Score (ES) | -0.25890842 |
| Normalized Enrichment Score (NES) | -1.7166494 |
| Nominal p-value | 0.0057636886 |
| FDR q-value | 0.09869635 |
| FWER p-Value | 0.517 |
Table: GSEA Results Summary

  

Fig 1: Enrichment plot: GO\_PERINUCLEAR\_REGION\_OF\_CYTOPLASM      
 Profile of the Running ES Score & Positions of GeneSet Members on the Rank Ordered List

  

| SYMBOL | TITLE | RANK IN GENE LIST | RANK METRIC SCORE | RUNNING ES | CORE ENRICHMENT || 1 | 10207 | INADL | 73 | 0.851 | -0.0293 | No |
| 2 | 92140 | MTDH | 93 | 0.801 | -0.0202 | No |
| 3 | 5967 | REG1A | 100 | 0.788 | -0.0017 | No |
| 4 | 7248 | TSC1 | 158 | 0.718 | -0.0231 | No |
| 5 | 10206 | TRIM13 | 161 | 0.716 | -0.0038 | No |
| 6 | 3303 | HSPA1A | 206 | 0.669 | -0.0169 | No |
| 7 | 3304 | HSPA1B | 207 | 0.669 | 0.0025 | No |
| 8 | 55275 | VPS53 | 278 | 0.609 | -0.0317 | No |
| 9 | 324 | APC | 281 | 0.607 | -0.0155 | No |
| 10 | 538 | ATP7A | 305 | 0.588 | -0.0155 | No |
| 11 | 26259 | FBXW8 | 318 | 0.583 | -0.0075 | No |
| 12 | 6715 | SRD5A1 | 325 | 0.578 | 0.0049 | No |
| 13 | 8125 | ANP32A | 326 | 0.578 | 0.0216 | No |
| 14 | 1203 | CLN5 | 358 | 0.562 | 0.0150 | No |
| 15 | 23242 | COBL | 426 | 0.516 | -0.0196 | No |
| 16 | 4771 | NF2 | 428 | 0.516 | -0.0054 | No |
| 17 | 5295 | PIK3R1 | 467 | 0.493 | -0.0192 | No |
| 18 | 51112 | TRAPPC12 | 504 | 0.471 | -0.0322 | No |
| 19 | 81031 | SLC2A10 | 539 | 0.453 | -0.0443 | No |
| 20 | 7099 | TLR4 | 541 | 0.451 | -0.0319 | No |
| 21 | 5567 | PRKACB | 595 | 0.426 | -0.0588 | No |
| 22 | 11226 | GALNT6 | 659 | 0.397 | -0.0939 | No |
| 23 | 56897 | WRNIP1 | 715 | 0.367 | -0.1240 | No |
| 24 | 9131 | AIFM1 | 728 | 0.360 | -0.1224 | No |
| 25 | 3323 | HSP90AA4P | 793 | 0.329 | -0.1602 | No |
| 26 | 7037 | TFRC | 824 | 0.303 | -0.1736 | No |
| 27 | 3673 | ITGA2 | 858 | -0.264 | -0.1904 | No |
| 28 | 4502 | MT2A | 863 | -0.277 | -0.1853 | No |
| 29 | 26031 | OSBPL3 | 870 | -0.281 | -0.1816 | No |
| 30 | 50640 | PNPLA8 | 881 | -0.296 | -0.1804 | No |
| 31 | 10241 | CALCOCO2 | 954 | -0.373 | -0.2229 | No |
| 32 | 81544 | GDPD5 | 961 | -0.381 | -0.2163 | No |
| 33 | 4864 | NPC1 | 964 | -0.384 | -0.2066 | No |
| 34 | 53349 | ZFYVE1 | 1018 | -0.417 | -0.2337 | No |
| 35 | 80315 | CPEB4 | 1053 | -0.448 | -0.2459 | Yes |
| 36 | 1191 | CLU | 1056 | -0.450 | -0.2343 | Yes |
| 37 | 5889 | RAD51C | 1059 | -0.451 | -0.2227 | Yes |
| 38 | 64423 | INF2 | 1067 | -0.456 | -0.2147 | Yes |
| 39 | 3918 | LAMC2 | 1088 | -0.466 | -0.2160 | Yes |
| 40 | 1889 | ECE1 | 1091 | -0.468 | -0.2039 | Yes |
| 41 | 857 | CAV1 | 1098 | -0.475 | -0.1945 | Yes |
| 42 | 8864 | PER2 | 1109 | -0.483 | -0.1879 | Yes |
| 43 | 2706 | GJB2 | 1115 | -0.490 | -0.1774 | Yes |
| 44 | 5359 | PLSCR1 | 1146 | -0.511 | -0.1848 | Yes |
| 45 | 27074 | LAMP3 | 1158 | -0.522 | -0.1777 | Yes |
| 46 | 1540 | CYLD | 1162 | -0.530 | -0.1646 | Yes |
| 47 | 56180 | MOSPD1 | 1186 | -0.558 | -0.1654 | Yes |
| 48 | 26471 | NUPR1 | 1195 | -0.570 | -0.1548 | Yes |
| 49 | 26999 | CYFIP2 | 1234 | -0.611 | -0.1652 | Yes |
| 50 | 1956 | EGFR | 1266 | -0.657 | -0.1691 | Yes |
| 51 | 8796 | SCEL | 1267 | -0.658 | -0.1500 | Yes |
| 52 | 1116 | CHI3L1 | 1339 | -0.777 | -0.1800 | Yes |
| 53 | 4734 | NEDD4 | 1369 | -0.856 | -0.1767 | Yes |
| 54 | 25819 | NOCT | 1374 | -0.891 | -0.1538 | Yes |
| 55 | 10397 | NDRG1 | 1380 | -0.925 | -0.1306 | Yes |
| 56 | 64764 | CREB3L2 | 1382 | -0.930 | -0.1044 | Yes |
| 57 | 8867 | SYNJ1 | 1387 | -0.979 | -0.0790 | Yes |
| 58 | 2040 | STOM | 1389 | -0.988 | -0.0510 | Yes |
| 59 | 81573 | ANKRD13C | 1393 | -1.049 | -0.0228 | Yes |
| 60 | 2591 | GALNT3 | 1403 | -1.195 | 0.0052 | Yes |
Table: GSEA details [plain text format]

  

Fig 2: GO\_PERINUCLEAR\_REGION\_OF\_CYTOPLASM      
 Blue-Pink O' Gram in the Space of the Analyzed GeneSet

  

Fig 3: GO\_PERINUCLEAR\_REGION\_OF\_CYTOPLASM: Random ES distribution      
 Gene set null distribution of ES for **GO\_PERINUCLEAR\_REGION\_OF\_CYTOPLASM**
